# Adverse events of recreational cannabis use during pregnancy reported to the French Addictovigilance Network between 2011 and 2020

**DOI:** 10.1038/s41598-022-19197-2

**Published:** 2022-10-03

**Authors:** Emilie Bouquet, Céline Eiden, Bernard Fauconneau, Charlotte Pion, French Addictovigilance Network (FAN), Carton Louise, Chevalier Cécile, Daveluy Amélie, Fournier-Choma Christine, Heredia Julie, Istvan Marion, Jouanjus Emilie, Peyrière Hélène, Pochard Liselotte, Revol Bruno, Savignat Véronique, Tournebize Juliana, Stéphanie Pain, Marie-Christine Pérault-Pochat

**Affiliations:** 1 Department of Clinical Pharmacology, Addictovigilance Center, Poitiers University Hospital, 2 rue de la Milétrie, 86 021 Poitiers, France; 2 Experimental and Clinical Neurosciences Laboratory, INSERM U-1084, University of Poitiers, Poitiers, France; 3 Addictovigilance Center, Montpellier University Hospital, Montpellier, France; 4 French National Agency for Medicines and Health Products Safety (ANSM), Saint-Denis, France; 5 Addictovigilance Center, Lille University Hospital, Lille, France; 6 Addictovigilance Center, Lyon University Hospital, Lyon, France; 7 Addictovigilance Center, Bordeaux University Hospital, Bordeaux, France; 8 Addictovigilance Center, Clermont-Ferrand University Hospital, Clermont-Ferrand, France; 9 Addictovigilance Center, AP-HP University Hospital, Paris, France; 10 Addictovigilance Center, Nantes University Hospital, Nantes, France; 11 Addictovigilance Center, Toulouse University Hospital, Toulouse, France; 12 Addictovigilance Center, AP-HM University Hospital, Marseille, France; 13 Addictovigilance Center, Grenoble University Hospital, Grenoble, France; 14 Addictovigilance Center, Caen University Hospital, Caen, France; 15 Addictovigilance Center, Nancy University Hospital, Nancy, France

**Keywords:** Risk factors, Health care, Public health

## Abstract

Cannabis is the main illicit psychoactive substance used by pregnant women in France. The aim of the present national survey was to describe adverse events (AEs) of recreational cannabis use during pregnancy reported to the French Addictovigilance Network (FAN). Spontaneous reports (SRs) of AEs related to recreational cannabis use during pregnancy were collected by the FAN between 01/01/2011 and 31/01/2021 (excluding cannabidiol and synthetic cannabinoids). Over the study period, 160 SRs involved cannabis use alone or in association with tobacco (59% of all SRs) which increased. Among the 175 maternal AEs, the most commons were psychiatric AEs experienced by 96 (64.9%) women, in particular cannabis use disorders (n = 89, 60.1%), dependence (n = 54, 36.5%) and abuse (n = 21, 14.2%). Among the 57 fetal AEs, the most common were heart rhythm disorders that affected 25 (16.9%) fetuses and intrauterine growth restriction (IUGR) (n = 20, 13.5%). Among the 140 neonatal AEs, the most common were IUGR experienced by 39 (26.3%) newborns and prematurity (n = 32, 21.6%). Twelve cases of congenital malformations were observed and 4 intrauterine/neonatal deaths. Furthermore, some of these AEs (n = 13) were unexpected. Cannabis use during pregnancy has problematic consequences for both mothers and infants who need close monitoring.

## Introduction

Cannabis is the main illicit psychoactive substance used by women of childbearing age in France. In 2017, 4% of women aged 18 to 34 years reported consuming cannabis at least ten times a month^1^.

Cannabis contains over 120 cannabinoids, the best characterized is delta-9-tetrahydrocannabinol (THC). THC crosses the placenta during the embryonic and fetal periods but there is still scarce knowledge about the pharmacokinetic properties of cannabis during pregnancy. Its distribution in the fetal compartment has not yet been studied. It is detected in meconium, urine and hair of infants exposed in utero. Blackard et al. measured THC and 9-carboxy-THC simultaneous in cord blood and maternal blood and showed concentrations in cord blood 3 and 6 times lower than in the maternal blood^2^. Furthermore, Boskovic et al. measured different THC content in the meconium and the hair of dizygotic twins, which suggested that some fetal and placental factors may modulate the fetal exposure^3^.

Very few data on the prevalence of cannabis use and its complications are available in France. According to the last national perinatal survey 2016, 2.1% of women interviewed in postpartum care reported using cannabis during pregnancy, almost half more than three times a month^4^. Furthermore, the French pharmaco-epidemiological survey OPPIDUM (Observation of illegal drugs and misuse of psychotropic medications) showed a significant rise in cannabis consumption among pregnant women seen in specialized addiction care centers from 1.2% in 2005–2006 to 17.8% in 2017–2018^5^.

In France, the potential for abuse and dependence of psychoactive substances is assessed by the French Addictovigilance Network (FAN) in collaboration with the National Agency for the Safety of Medicines and Health Products (Agence Nationale de Sécurité du Médicament et des Produits de Santé). This surveillance system regulated by law is based on spontaneous reporting by healthcare professionals, collaborators and patients completed by specific pharmaco-epidemiological surveillance programs^6–8^.

A previous national survey showed a wide variety of adverse events (AEs) of recreational cannabis use reported to the FAN over the period 2012–2017, including some perinatal AEs (n = 55, 1.7% of all AEs). They were mainly dependence (21.8% of the perinatal AEs), IUGR (21.8% of the perinatal AEs), prematurity/premature rupture of membranes (n = 7, 12.7% of perinatal AEs)^9^. Five of them were unexpected: three cases of congenital malformations (cardiopathy, horse kidney and duodenal atresia), one case of oligohydramnios and one case of bartholinitis^9^. Given the recent increase in Addictovigilance signals, an overview was necessary.

The aim of the present national survey was to described AEs of recreational cannabis use during pregnancy by exploring cases reported to the FAN the ten last years.

## Methods

According to French law, any serious cases of abuse and dependence involving psychoactive substances (with the exception of alcohol and tobacco) must be reported by health professionals to the FAN. The FAN consists of 13 addictovigilance centers implemented in university hospital throughout France. Practitioners from the FAN are pharmacologists who answer to health professionals about the risks of psychoactive substances use, who investigate spontaneous reports (SRs) to complete and analyze them in order to identify or confirm public health signals. After their analysis, SRs are recorded over time in the addictovigilance database by respecting the anonymity of the patients and the notifiers.

We identified all SRs of AEs related to recreational cannabis use during pregnancy collected by the FAN between January 1, 2011 and January 31, 2021 (excluding cannabidiol and synthetic cannabinoids).

All cases were reviewed by an expert pharmacologist. The addictovigilance database was approved by the French Data Protection Agency (CNIL), and data were recorded anonymously, therefore there was no direct clinical responsibility for patients, no required informed consent and no research ethics committee approval.

Events were classified in three categories: maternal, fetal and neonatal events. They were coded using the Medical Dictionary for Regulatory Activities (MedDRA).

According to the Diagnostic and Statistical Manual of Mental Disorders (DSM-IV) and the International Classification of Diseases and Related Health Problems (ICD-10), abuse was defined as an excessive and intentional use, occasional or persistent with harmful physical and/or psychological effects and dependence as a craving, an inability to reduce or control cannabis use despite health problems caused by its use, a withdrawal syndrome, a phenomenon of tolerance resulting in a consumption of larger amounts that were intended or spending a lot of time to get, use or recover from effects^10,11^. In mid-2013, DSM-V replaced DSM-IV and abuse and dependence were combined under the terminology cannabis use disorders^12^. Thus, the coded terms were abuse and dependence until mid-2013, and then cannabis use disorders.

Problematic use was defined by a punctual, regular or chronic use resulting in negative medical and social consequences. It differs from abuse because there is not necessary excessive consumption.

Intrauterine growth restriction was defined as a birth weight for gestational age less than the 10th percentile.

Events were considered unexpected if they had been described in the literature only rarely or not at all.

Exposures were classified in three categories: during the first, the second and the third trimester of pregnancy. When consumption was related in number of joints or number of times a day during pregnancy, it was considered that cannabis was consumed throughout pregnancy.

In some cases, toxicological tests were performed in the context of medical care.

The analysis of congenital malformations focused on cases exposed to cannabis at least in the first trimester of pregnancy. The analysis of neonatal withdrawal syndromes focused on cases exposed to cannabis at least in the third trimester of pregnancy.

To describe the evolution of reports, the percentage of all SRs on cannabis use during pregnancy among the total number of national SRs was calculated for each year. The rate of reported cases per 100,000 live births was estimated using the French national institute of statistics and economic studies (Institut National de la Statistique et des Etudes Economiques—INSEE) data on live births^13^.

The characteristics of cannabis pregnant users, cannabis use during pregnancy and the main AEs were described and then compared between two groups exposed to “cannabis alone or in association with tobacco” and “cannabis and others psychoactive substances/medicinal products”. The means and frequencies were reported for quantitative and qualitative variables, respectively. Chi-square test and Student test were performed to compare the two groups when relevant, using the statistical software R (version 3.6.2, R Core Team, 2019).

To assess AEs related to cannabis use during pregnancy, we focused only on cases involving cannabis alone or in association with tobacco due to their frequent concomitant use. Those involving concomitant use of alcohol, other substances or drugs such as cocaine or methadone were excluded because of the risk of bias related to their use.

## Results

### Characteristics of SRs

A total of 271 SRs of one or more events involving cannabis use during pregnancy were included in the study. They occurred in 242 pregnancies, some of them concerning both the mother and the fetus and/or the newborn. The proportion of SRs involving cannabis use during pregnancy represented a small proportion of the total national SRs. It significantly increased from 0.16% in 2011 to 1.10% in 2020 (p < 0.001). This corresponds to a notified rate multiplied by 7 whereas the total national number of SRs increased by around 2.5-fold over the same period (data not shown). The estimated reporting rate of SRs related to cannabis use during pregnancy per 100,000 live births increased from 0.49 reports per 100,000 live births in 2011 to 8.38 reports per 100,000 live births (p < 0.0001).

### Characteristics of pregnant women

The mean age of pregnant women was 27.8 years (range 16–44 years) and three were minors. A history of substance abuse was reported in 59.5% of them and a psychiatric history in 27.7% of them (Table 1).

**Table 1:** Characteristics of cannabis pregnant users.

Maternal characteristics	All (N = 242)		Cannabis ± tobacco (N = 148)		Cannabis + other psychoactive substances/medicinal products (N = 94)		p*
	N	%	N	%	N	%	
**Age (years)**	200	82.6	132	89.2	68	72.3	
< 18 years	3	1.2	3	2.0	0	0.0	
Mean	27.8		26.6		30.2		** < 0.001**
Range	16–44		16–40		18–44		
**History of substance use and dependence**	144	59.5	86	58.1	58	61.7	**0.54**
**Psychiatric history**	67	27.7	32	21.6	35	37.2	**0.01**
**Parity**							
Nulliparous	22	9.1	13	8.8	9	9.6	**0.84**
Multiparous	30	12.4	21	14.2	10	10.6	**0.29**
Unknown	190	78.5	114	77.0	76	80.9	
**History of spontaneous miscarriage**	15	6.2	9	6.1	6	6.4	**0.92**
**History of abortion**	35	14.5	23	15.5	12	12.8	**0.55**
**Twin pregnancy**	5	2.1	4	2.7	1	1.1	

*p value derived from Chi-square test for comparison of proportions and Student test for comparison of means.

Significant values are in bold.

The characteristics of pregnant women who reported cannabis use alone or in association with tobacco were different from those who used cannabis and other psychoactive substances/medicinal products: they were younger (mean age: 26.6 versus 30.2 years, p < 0.001) and had fewer psychiatric history (21.6% versus 37.2%, p = 0.01).

### Characteristics of cannabis use during pregnancy

Table 2 shows the characteristics of cannabis use during pregnancy. When the route of administration was specified, it was always inhaled (59.9%) (Table 2). Cannabis use in the first trimester of pregnancy was chronic in 64.5% of cases (daily in 58.3% of cases and greater than ten times a day in 9.5% of cases). Pregnant women who used cannabis alone or in association with tobacco reported more frequently a chronic use in the first trimester of pregnancy (69.6% versus 56.4% among those who reported cannabis and other substances/medicinal products use), in particular a daily use (63.5% versus 50.0% among those who reported cannabis and other substances/medicinal products use, p = 0.04) (Table 2).

**Table 2:** Characteristics of cannabis use during pregnancy.

	All (N = 242)		Cannabis ± tobacco (N = 148)		Cannabis + other psychoactive substances/medicinal products (N = 94)		p*
	N	%	N	%	N	%	
**Mode of use**							
Inhaled	145	59.9	97	65.5	48	51.1	**0.02**
Unknown	97	40.1	51	34.5	46	48.9	
**Cannabis use**							
**In the first trimester**	N = 242		N = 148		N = 94		
Yes	238	98.3	146	98.6	92	97.9	**0.95**
Unknown	4	1.7	2	1.4	2	2.1	
Daily	141	58.3	94	63.5	47	50.0	**0.04**
≥ 10 Times/day	23	9.5	11	7.4	12	12.8	**0.17**
Concomitant use of tobacco							
Yes	114	47.1	66	44.6	48	51.1	**0.39**
Unknown	128	52.9	82	55.4	46	48.9	
**In the second trimester**	N = 241		N = 147		N = 94		
Yes	225	93.4	140	95.2	85	90.4	**0.14**
No	2	0.8	0	0.0	2	2.1	
Unknown	14	5.8	7	4.8	7	6.4	
Daily	133	55.2	90	60.8	43	45.7	**0.02**
≥ 10 Times/day	19	7.9	9	6.1	10	10.6	**0.20**
Concomitant use of tobacco							
Yes	112	46.5	65	43.9	47	50.0	**0.45**
Unknown	129	53.5	83	56.1	46	48.9	
**In the third trimester**	N = 241		N = 147		N = 94		
Yes	219	90.9	133	90.5	86	91.5	**0.79**
No	4	1.7	1	0.7	3	3.2	
Unknown	18	7.5	13	8.8	4	4.3	
Daily	127	52.7	86	58.1	41	43.6	**0.02**
≥ 10 Times/day	18	7.5	8	5.4	10	10.6	**0.21**
Concomitant use of tobacco							
Yes	109	45.2	64	43.2	45	47.9	**0.60**
No	2	0.8	1	0.7	1	1.1	
Unknown	130	53.9	83	56.1	47	50.0	
**Stopping use during pregnancy**	15	6.2	7	4.7	8	8.5	**0.36**
In the first trimester	6	2.5	0	0.0	6	6.4	
In the second trimester	3	1.2	2	1.4	1	1.1	
In the third trimester	6	2.1	5	3.4	1	1.1	
**Decreasing use during pregnancy**	25	10.3	18	12.2	7	7.4	**0.32**
**Increasing use during pregnancy**	3	1.2	1	0.7	2	2.1	
**Test positive for cannabis**	35	14.5	11	7.4	25	26.6	** < 0.001**

*p value derived from Chi-square test for comparison of proportions.

Significant values are in bold.

Few pregnant women (6.2%) stopped consuming cannabis during pregnancy (2.5% in the first trimester, 1.2% in the second trimester and 2.1% in the third trimester). A decrease in cannabis use was reported by 10.3% of them and an increase in cannabis use by 1.2% of them (Table 2).

A concomitant use of tobacco was reported in 47.1% of cases in the first trimester, in 46.5% of cases in the second trimester and in 45.2% of cases in the third trimester (Table 2). A concomitant use of alcohol was mentioned in 12.4% of cases in the first trimester, in 8.7% of cases in the second trimester and in 7.5% of cases in the third trimester. Concomitant uses of other psychoactive substances were mainly cocaine (12.8% in the first trimester, 9.1% in the second trimester, 9.5% in the third trimester) and heroin (5% in the first trimester, 5.8% in the second trimester and 4.6% in the third trimesters). Concomitant uses of medicinal products were mainly opioid substitution treatments (13.6% in the first trimester, 14.1% in the second trimester, 13.7% in the third trimester), in particular methadone (9.9% in the first trimester, 10% in the second trimester, 9.5% in the third trimester). Benzodiazepines and benzodiazepine-like agents were placed in second position (9.1% in the first and second trimesters, 10% in the third trimester) and then antipsychotics (2.5% in the first and second trimesters, 2.9% in the third trimester), serotonin reuptake inhibitors (2.1% in the first trimester, 1.7% in the second and third trimesters) and anticonvulsants (1.2% in the first trimester, 0.8% in the second and third trimesters) (data not shown).

In 35 (14.5%) of cases, a toxicological test was positive for cannabis in the pregnant women or newborns. They were less frequent in women who reported cannabis use alone or in association with tobacco than in those who reported cannabis and other substances/medicinal products use (Table 2).

Among the 38 pregnant women (15.7%) who described the reasons for their cannabis consumption, 28 (73.7%) indicated consuming it for its anxiolytic/sedative effects, 4 (10.5%) for occupational effects, 3 (7.9%) ex-aequo for hypnotic effects, disinhibition, pleasure, motivation help, recreational use, as a substitute of alcohol and 2 (5.3%) for its anti-emetic effect (data not shown).

### Adverse events related to cannabis use alone or in association with tobacco

Over the study period, 160 of the 271 SRs involved cannabis use alone or in association with tobacco and they concerned 148 pregnancies (Table 3).

**Table 3:** Main AEs related to cannabis use during pregnancy in women and fetus and/or neonates.

	All (N = 242 pregnancies)	Cannabis ± tobacco (N = 148 pregnancies)	Cannabis + other psychoactive substances/medicinal products (N = 94 pregnancies)	p*
**In women**				
Psychiatric	140 (57.9%)	96 (64.9%)	44 (46.8%)	**0.006**
Use disorder	123 (50.8%)	89 (60.1%)	39 (41.5%)	**0.007**
Dependence	79 (32.6%)	54 (36.5%)	25 (26.6%)	**0.11**
Abuse	26 (10.7%)	21 (14.2%)	5 (5.3%)	**0.03**
Problematic use	16 (6.6%)	6 (4.0%)	5 (5.3%)	**0.89**
Threat of premature birth	18 (7.4%)	13 (8.8%)	5 (5.3%)	**0.32**
Premature rupture of membranes	15 (6.2%)	12 (8.1%)	3 (3.2%)	**0.12**
**In fetus and/or neonates**				
IUGR in fetus and/or neonate	78 (32.2%)	48 (32.4%)	30 (31.9%)	**0.93**
Prematurity	50 (20.7%)	32 (21.6%)	18 (19.1%)	**0.64**
Withdrawal syndrome	45 (18.6%)	11 (8.1%)	34 (36.2%)	** < 0.001**
Fetal heart rhythm disorders	34 (14.0%)	25 (16.9%)	10 (10.6%)	**0.18**
Congenital malformations in fetus and/or neonate	19 (7.8%)	12 (8.1%)	7 (7.4%)	**0.85**
Apgar ≤ 7 at 1 min	18 (7.4%)	11 (7.4%)	7 (7.4%)	**1.0**
Respiratory failure	16 (6.6%)	7 (4.7%)	9 (9.6%)	**0.14**
Intrauterine fetal death, neonatal death	6 (2.5%)	4 (2.7%)	2 (2.1%)	**0.57**

*p value derived from Chi-square test for comparison of proportion.

Significant values are in bold.

The number of maternal AEs (n = 175) related to cannabis use alone or in association with tobacco increased significantly from 0 in 2011 to 5 (2.9% of AEs, 95% CI 0.4–5.3%) in 2012 and 25 (13.7%, 95% CI 8.6–18.8%) in 2020 (data not shown). Some of them were AEs usually described in cannabis users. Women who reported cannabis use alone or in association with tobacco presented more frequently psychiatric AEs than those who reported cannabis and other psychoactive substances/medicinal products use (n = 96, 64.9% versus n = 44, 46.8%, p = 0.006) (Table 3). They experienced cannabis use disorders (n = 89, 60.1%), dependence (n = 54, 36.5%), abuse (n = 21, 14.2%) and problematic use (n = 6, 4.0%) (Table 3). Furthermore, five women (3.4%) suffered from cannabinoid hyperemesis syndrome (Table S1). The other most common AEs were threat of premature membranes that were experienced by 13 (8.8%) women and premature rupture of membranes experienced by 12 (8.1%) women (Table 3).

Some women presented unexpected AEs (n = 8, 5.4%): anhydramnios/oligohydramnios (n = 6, 4.0%), preeclampsia (n = 1, 0.7%) and bartholinitis (n = 1, 0.7%) (Table S1).

The number of fetal AEs (n = 57) related to cannabis use alone or in association with tobacco significantly increased from 0 in 2011 to 1 in 2012, 5 (8.8%, 95% CI 1.4–16.1) in 2015 and 23 (40.3%, 95% CI 27.6–53.1) in 2020. Fetus presented most often heart rhythm disorders (n = 25, 16.9%), IUGR (n = 20, 13.5%), congenital malformations diagnosed prenatally (n = 8, 5.4%), and two (1.3%) intrauterine fetal deaths (Table S1).

The number of neonatal AEs (n = 140) related to cannabis use alone or in association with tobacco significantly increased from 0 in 2011 to 4 (2.8%, 95% CI 0.1–5.6) in 2012 and 55 (39.3%, 95% CI 31.2–47.4) in 2020. Infants presented mainly IUGR (n = 39, 26.3%, some of them diagnosed prenatally), prematurity (n = 32, 21.6%), Apgar score ≤ 7 at 1 min (n = 11, 7.4%), withdrawal syndromes (n = 11, 7.4%), congenital malformations (n = 9, 6.1%) and respiratory failure (n = 7, 4.7%). Fifteen newborns (10.1%) were admitted in intensive care. Furthermore, two (1.3%) neonatal deaths were reported in very preterm infants: in one case it was a twin pregnancy and the cause of death was unknown, in the other case, the newborn had intraventricular hemorrhage, leukomalacia, obstructive hydrocephalus, pulmonary arterial hypertension, acute renal failure, congenital malformation to type of arthrogyrosis, inches of bilateral adductus, low set ears, hypospadias in a context of prolonged anhydramnios and he died on the eighth day of life (Table S1). Some infants presented unexpected AEs (n = 5, 3.4%): sacrococcygeal teratoma (n = 1, 0.7%), crossed renal ectopia (n = 1, 0.7%), horseshoe kidney (n = 1, 0.7%), duodenal atresia (n = 1, 0.7%) and hypospadias (n = 1, 0.7%).

### Focus on congenital malformations

Twelves of the nineteen cases of congenital malformations occurred in fetuses/newborns whose mothers reported cannabis use alone or in association with tobacco (8.1% of pregnancies). They were limb malformations (n = 4, arthrogyrosis, inches of bilateral adductus in a context of anhydramnios, bilateral equine varus feet, short femur), microcephaly (n = 3, including one case with dolicocephalia), kidney malformations (n = 2, crossed renal ectopia, horseshoe kidney), congenital malformation of the face (n = 2, low set ears, philtrum elongated with thin upper lip and small chin), hypospadias (n = 1), sacrococcygeal teratoma (n = 1), cardiopathy (n = 1), omphalocele (n = 1) and duodenal atresia (n = 1) (Table 4).

**Table 4:** Congenital malformations in fetus and/or neonates exposed in utero to cannabis alone or in association with tobacco.

Type of congenital malformation	Other AEs	Sex	Maternal age (year)
Arthrogyrosis, inches of bilateral adductus, low set ears, hypospadias	Premature rupture of membranes Anhydramnios Prematurity Apgar ≤ 7 at 1 min Intrauterine growth restriction Respiratory failure Intraventricular haemorrhage (grade 4) and leucomalacia Obstructive hydrocephalus Pulmonary arterial hypertension Acute renal failure Neonatal death at D8	M	20
Microcephalia with dolichocephalia	Oligohydramnios Prematurity Intrauterine growth restriction Enteropathy	F	24
Filtrum elongated with thin upper lip and small chin, microcephalia (antenatal diagnosis: facial dysmorphia and thymus hypoplasia)	Maternal cannabis dependence Pre-eclampsia Prematurity Intrauterine growth restriction Respiratory failure, bradycardia, hypotonia, thrombopenia, neonatal jaundice, hypoglycemia, intraventricular hemorrhage with ventriculomegaly	M	36
Bilateral equine varus feet	Maternal cannabis dependence	M	27
Sacrococcygeal teratoma (antenatal diagnosis)	Maternal cannabis use disorder Fetal heart rhythm disorder	Unknown	37
Microcephalia (antenatal diagnosis)	Intrauterine growth restriction	F	Unknown
Crossed renal ectopia (antenatal diagnosis: left renal agenesis)	Maternal behavioural disorder Premature rupture of membranes Anhydramnios Prematurity Intrauterine growth restriction Respiratory failure Hyaline membrane disease Anemia Retinopathy Placental subchorionic thrombosis	F	21
Horseshoe kidney (antenatal diagnosis: left kidney not seen)	Maternal cannabis abuse Fetal heart rhythm disorder Apgar ≤ 7 at 1 min	M	24
Duodenal atresia	Maternal cannabis abuse Premature rupture of membranes	F	31
Omphalocele (antenatal diagnosis)	Maternal cannabis dependence	Unknown	22
Cardiopathy (antenatal diagnosis)	Maternal cannabis use disorder and withdrawal syndrome Maternal gastric pain Maternal vomiting	Unknown	21
Short femur (antenatal diagnosis)	Maternal cannabis abuse Threat of premature birth Reduction in the fetal heart rate Intrauterine growth restriction	Unknown	23

*D* day, *M* male, *F* female.

Maternal age was less than 35 years old in 8 cases (66.7%). Cannabis was consumed during the whole pregnancy, most often regularly (when this data was available), nevertheless in two cases it was punctual (data not shown). Congenital malformations were associated with IUGR in 6 cases (4.0% of pregnancies exposed to cannabis alone or in association with tobacco), and with anhydramnios/oligohydramnios in 3 cases (2% of pregnancies exposed to cannabis alone or in association with tobacco). In most of the cases, several congenital anomalies were present without however being able to define a specific pattern of malformation (Table 4).

## Discussion

The proportion of SRs involving cannabis use during pregnancy reported to the FAN was low and represented a small proportion of the total national SRs. It increased sevenfold between 2010 and 2020 whereas the total national number of SRs increased by around 2.5-fold over the same period and the reporting rate in France of all AEs related to cannabis use alone or in association with tobacco and/or alcohol tripled between 2012 and 2017^9^. This increase may result from a rise in the prevalence of cannabis use during pregnancy, from a normalization of cannabis use leading pregnant women to confess it or because pregnant women were more likely to be questioning about their cannabis use.

The characteristics of women who used cannabis alone or in association with tobacco were different from those who were poly-consumers of psychoactive substances because they were younger, had less psychiatric history and reported more frequently chronic cannabis use.

The maternal events related to cannabis use alone or in association with tobacco were in the first place psychiatric, in particular use disorders and dependence, which is consistent with previous results observed more generally in cannabis users^9^. These conditions may complicate the management of the pregnancy, in particular in women with past-history of sustained daily cannabis use. Furthermore, nine maternal AEs were unexpected such as anhydramnios/oligohydramnios, pre-eclampsia/eclampsia and bartholinitis. The last two cases have always been mentioned in our previous study^9^.

Fetal events related to cannabis use alone or in association with tobacco were mainly fetal heart rhythm disorders, intrauterine growth restriction and congenital malformations diagnosed prenatally. It is also noteworthy that two cases of intrauterine fetal deaths were also reported.

Neonatal events related to cannabis use alone or in association with tobacco were mainly intrauterine growth restriction and prematurity, which was expected. A previous study more than ten years ago had shown an increased risk of growth restriction in infants whose mothers used cannabis during pregnancy compared with those whose mothers smoked tobacco^14^ and an increased risk of preterm birth in pregnant women who used cannabis, notably among those who also smoke tobacco^15^. Other frequent effects were Apgar score ≤ 7 at 1 min, withdrawal syndromes, congenital malformations and respiratory failure. Five AEs were unexpected such as sacrococcygeal teratoma, crossed renal ectopia, horseshoe kidney, duodenal atresia and hypospadias and three of them have always been described in our previous study^9^. In most of cases, several congenital malformations were associated without being able to define a specific pattern of malformation. Furthermore, two neonatal deaths were reported in very preterm infants.

France is the only European country with a national specifically proactive addictovigilance surveillance system to evaluate the potential of abuse and dependence of psychoactive substances. The present study is the first to give a national overview of AEs related to recreational cannabis use alone or in association with tobacco during pregnancy these ten last years. The choice of excluding cases related to cannabis use in association with other substances was intended to provide an overall description of the potential events related only to the use of cannabis frequently associated with tobacco smoking during pregnancy.

This study based on spontaneous reporting is subject to underreporting^16^. Furthermore, cannabis use during pregnancy is frequently underreported by pregnant women due to the stigma associated with it. Consequently, these findings do not reflect all AEs related to cannabis use during pregnancy in general population. It cannot be excluded that birth anomalies were likely to reflect differential reporting of cannabis use in women whose infant suffered from them. But these results provide hypotheses that need to be tested in large representative samples of women who have been asked about their cannabis use before birth and confirmed by biomarkers of cannabis use. In addition, one addictovigilance center collected nearly 40% of SRs resulting from hospital consultations specialized in addictology that is likely to induce a bias in the severity of the effects. Pregnant women were more likely to be questioning by these practitioners about their cannabis use and to report it, which in turn can increase the risk of reporting bias, thus making it difficult to interpret the increased reporting of AEs. This analysis of SRs did not allow to compare cases to those that occurred in pregnant women not exposed to cannabis so that causality could not be assessed. Unfortunately, some of the data that were collected from spontaneous notifications were likely to be missing and tobacco use was not always provided.

The endocannabinoid system is involved in different stages of reproduction and for early pregnancy and maintenance^17^. Thus an imbalance secondary to the use of cannabis at key moments is likely to induce deleterious effects. Moreover, the mean THC content of cannabis products has increased over the past 10 years worldwide^18,19^. In France, it rose for cannabis resin from 10% in 2009 to 28% in 2019 and for herbal cannabis, it maintained around 12% in 2019 compared to 7% in 2009^18,20^. This may result in an increased in utero exposure to THC and risk of AEs.

When women reported consuming cannabis alone, it was difficult to absolutely exclude the role of tobacco due to the frequent concomitant use, notably by inhalation. Tobacco is also known to induce perinatal adverse effects, notably preterm births and intrauterine growth restriction with a dose–response effect^21–23^. Kyrklund-Blomberg et al. reported an increased risk of prematurity by 1.2 [1.1–1.2] among pregnant women smoking 1 to 9 cigarettes/day and by 1.4 [1.3–1.5] among those smoking more than 10 cigarettes/day compared to nonsmoker pregnant women^21^. Another retrospective study showed an increased risk of prematurity by 1.41 [1.37–1.44] among cigarettes smoking pregnant women^22^. According to a meta-analysis of 210 studies, the rate of small for gestational age was nearly twice in cigarette smoking pregnant women (pooled aOR 1.95 [1.76–2.16]), the rate of shorter length was lower (pooled mean difference = 0.43 [0.41–0.44]), as well as smaller head circumference (pooled mean difference = 0.27 [0.25–0.29])^23^. Maternal smoking appears to be associated with a lower risk of preeclampsia. A meta-analysis of 70 prospective studies reported that smoking pregnant women was inversely associated with the incidence of preeclampsia (RR = 0.67 [0.60–0.75])^24^. However, co-use of cannabis and tobacco seems to be associated with an increased risk of preeclampsia^25^. Furthermore, Shobeiri et al. showed that smoking during pregnancy was associated with a higher risk of placenta previa (OR 1.42 [1.30–1.54], RR = 1.27 [1.18–1.35])^26^.

Nevertheless, experimental data are particularly interesting because they allow to overcome biases observed in clinical studies and to analyze the effects of cannabis and/or THC alone. They have shown that cannabis and THC exposure during gestation resulted in reduced birth weight and increased embryonic and fetal mortality^27,28^. This may be explained by the role of cannabis on placental insufficiency^29^, lower placental GLUT1 expression that may decrease the availability of glucose which is the primarily energy source for the fetus^27^. Furthermore, THC reduces the invasion of extravillous trophoblasts in vitro HTR-8/SV neo cells in a spheroid invasion model^30,31^, which suggests its role in the occurrence of preeclampsia.

Regarding the six cases of anhydramnios/oligohydramnios, one had no other contributing factor than a chronic cannabis use (6 joints/day at the beginning of the pregnancy reduced at 3 joints/day). For the five other cases, premature rupture of membranes, intra-uterine growth restriction and renal congenital malformation were also present.

Indeed, in published studies about the risk of congenital malformations, it was very often difficult to ruled out the role of concomitant substances^32^. Cases reported in this study did not allow to conclude to an increased risk, but some of them were unexpected (sacrococcygeal teratoma, crossed renal ectopia, horseshoe kidney, duodenal atresia and hypospadias). To date no increased risk is retained for tobacco, even if a weak association is discussed for specific malformations such as facial clefts, musculoskeletal defects, limb reduction, malposition of the foot, gastrointestinal defects and cardiopathies^33^. However, experimental studies have shown epigenetic disruptions related to cannabis exposure during pregnancy^28,34^. Moreover, chronic THC exposure may decrease the level of plasma folic acid, which plays an important role in preventing congenital malformation, in particular neural tube defects but also cardiac malformations, urinary tract anomalies, oro-facial clefts and limb reductions^35^. Animal studies on teratogen risk showed congenital anomalies (limb and extremity defects, encephalocele, eventration of abdominal visceral) in 57% of descendants of pregnant rats exposed to intraperitoneal emulsion of resin (4.2 mg/kg/day from day 1 to day 6 of gestation)^36^ but they have not been confirmed later^37,38^.

## Conclusion

Cannabis use during pregnancy, alone or in association with tobacco, has problematic consequences for both mothers and children who need close monitoring. Some of the reported events in this study were unexpected, such as anhydramnios/oligohydramnios, preeclampsia, bartholinitis and some congenital malformations (sacrococcygeal teratoma, crossed renal ectopia, horseshoe kidney, duodenal atresia and hypospadias). Even if the risks of smoking during pregnancy are serious, they are well-documented for tobacco much less for cannabis. Interestingly, experimental studies have showed effects of cannabis and/or THC alone on fetal growth and mortality as well as on epigenetic disruption and decreased levels of folic acid.

In our study, few women have discontinued their cannabis use during pregnancy. In the light of the normalization of recreational cannabis use, it seems therefore important to inform women of childbearing age, pregnant women, health professionals and policy makers about the deleterious effects of exposure to cannabis during pregnancy.

## Supplementary Information

Supplementary Table S1.

## Data Availability

The datasets generated or analyzed during the current study are not publicly available due to restrictions. According to the French Laws (Articles R.5132-113, R5132-114) each case was recorded in the French Addictovigilance database, in an anonymous format and are under the authority of the French National Agency for Medicines and Health Products Safety (ANSM). The corresponding author will on request detail the restrictions and any conditions under which access to some data may be provided.
