# Supplementary material for: Adverse events of recreational cannabis use during pregnancy reported to the French Addictovigilance Network between 2011 and 2020
Source: Sci Rep. 2022 Oct 3;12:16509. doi: 10.1038/s41598-022-19197-2 (PMC9530152; doi:10.1038/s41598-022-19197-2)
Supplement: Supplementary file 1 — Supplementary Table S1. [file 41598_2022_19197_MOESM1_ESM.docx]

**Table S1**: AEs involving cannabis use alone or in association with tobacco reported in pregnant women, fetus and neonates

| **Maternal (N=175)** | **Fetal (N=57)** | **Neonatal (N=140)** |
| --- | --- | --- |
| Dependence (54)  Abuse (21)  Use disorders (14)  Threat of premature birth (13)  Premature rupture of membranes (12)  Anamnios, oligoamnios (6)  Problematic use (6)  Cannabinoid hyperemesis syndrome (5)  Asthenia (5)  Epigastralgia, vomiting (4)  Asthma, worsening of asthma, bronchitis (3)  Depressed mood, anhedonia, aboulia, apathy (2)  Agitation (2)  Anxiety (2)  Behavioral disorder (2) Faintness (2)  Metrorrhagia (2)  Placental vascular malperfusion with sub-chorial thrombosis (2)  Amotivational syndrome (1)  Clastic crisis (1)  Boulimia (1)  Impulsivity (1)  Sleep disorders (1)  Anorexia (1)  Placenta previa (1)  Placental abruption (1)  Preeclampsia (1)  Headache (1)  Intracranial hypertension (1)  Impairment memory (1)  Bartholinitis (1)  First degree burns secondary to hot showers (1)  Cholestasis (1)  Hypokaliemia (1)  Gestational diabetes (1)  Volontary interruption of pregnancy (1) | Fetal heart rhythm disorders (25) : slowdown/bradycardia (8), tachycardia after one joint (1), unspecified (16)  Intrauterine growth retardation, small for gestational age (20)  Prenataly congenital malformation* (8)  Doppler anomaly (2)  Intrauterine fetal death (2) | Intrauterine growth retardation, small for gestational age (39)  Prematurity (32)  Apgar ≤ 7 at 1 min (11)  Withdrawal syndrome (11)  Congenital malformation ** (9)  Respiratory failure (7)  Hypotonia, sedation (4)  Neonatal jaundice ᵼ (3)  Neonatal death *** ᵼ (2)  Hypoglycemia (2)  Anemia ᵼ (2)  Bronchopulmonary dysplasia, hyaline membrane disease ᵼ (2)  Neonatal bradycardia (2)  Pulmonary arterial hypertension (1)  Weak suck (1)  Anoxo-ischemic brain damage (1)  Intraventricular hemorrhage ᵼ (2)  Eyelid clonia (1)  Choking (1)  Cytolysis (1)  Enteropathy (1)  Polycythemia (1)  Thrombopenia (1)  Retinopathy of prematurity (1)  Patent ductus arteriosus (1)  Acute renal failure (1) |

*omphalocele ; fetal cardiopathy ; sacrococcygeal teratoma ; left kidney not seen ; left renal agenesis ; microcephalia ; short femur ; facial dysmorphia and thymus hypoplasia

** arthrogyrosis, inches of bilateral adductus, low set ears (prolonged anamnios), hypospadias ; microcephalus with dolichocephalia ; philtrum elongated, thin upper lip, small chin and microencephalia ; microcephalia ; bilateral equin varus feet ; sacrococcygeal teratoma ; crossed renal ectopia ; horseshoe kidney ; duodenal atresia

*** prolonged anamnios, intraventricular hemorrhage, leukomalacia, obstructive hydrocephalie, pulmonary arterial hypertension, acute renal failure and arthrogyrosis, inches of bilateral adductus, low set ears, hypospadias (described above) in a very preterm infants (29 weeks of gestation); very preterm infant 30 weeks of gestation (twin pregnancy)

ᵼ in premature infants
